# Health Personnel Improvement in the Implementation of Shariah's Ethical Code of Conduct in Tangerang Hospital, Indonesia

**DOI:** 10.1155/2022/5548840

**Published:** 2022-05-09

**Authors:** Wahyu Sulistiadi, Sri Rahayu, Meita Veruswati, Al Asyary

**Affiliations:** ^1^Department of Health Administration and Policy, Faculty of Public Health, Universitas Indonesia, Depok, Indonesia; ^2^Center for Educational and Community Services, Faculty of Public Health, Universitas Indonesia (P3M FKM-UI), Depok, Indonesia; ^3^Department of Public Health Science, Universitas Muhammadiyah Prof. Dr. HAMKA (UHAMKA), Jakarta, Indonesia; ^4^Department of Environmental Health, Faculty of Public Health, Universitas Indonesia, Depok, Indonesia

## Abstract

**Background:**

The Shariah hospital aims to provide health services inclusive of the appropriate code of ethics of Sharia, which exceeds the standard of service expected of conservative hospitals. Hospital care depends on the performance of health professionals. The study aimed to assess the relationship between the implementation of the ethical code of Shariah hospitals and the performance of health personnel.

**Methods:**

The study is a quantitative analysis that collects cross-sectional data from 119 health personnel respondents in one Shariah hospital in Indonesia. Data analysis was performed using structural equation modeling (SEM).

**Results:**

The implementation of the ethical code of Shariah hospitals, whether explicit or implicit, has a significantly positive influence on the performance and well-being of health personnel, which is evident from the *t* value of 4.31 (more substantial than the *t* value of 1.960). This implementation should run consistently and with the commitment of all parties.

**Conclusion:**

Such insight, in turn, can be counted as an input to an approach to health services, particularly in increasing the performance rates in hospital. This study is the first to provide new insight into discussion about Shariah hospital's code of conduct by presenting its beneficiary to not only improve health personnels' performance but also provide the inclusive health service for all religions and cultures which is essential in further study.

## 1. Background

In line with the development of civilization, that of the order of sociocultural society, and that of the progress of science and technology, especially in the fields of medicine and health, the hospital has evolved into an entity that requires an orientation of the various socioeconomist complexities it often encounters [1, 2]. In the history of Indonesia, hospital management is based on the principles of Sharia. Such hospitals are known as Shariah hospitals, which have been granted Sharia certification by the Indonesian Ulema Council [3].

The ethical codes of Shariah hospitals as well as that of hospitals in general are applied in operational hospitals [3]. For doctors and health workers, ethics means the obligation and responsibility to meet the expectations of the profession and the public. For the leader of the hospital, ethics should mean the obligation and responsibility specifically towards the patients, the health personnels, the profession, the government, and other levels not apparent to the public including health professional organizations and nongovernment organizations. The criteria of fair, honest, professional, and respectable practices also apply to other officers in the hospital. The ethical code of a hospital consists of ethical practices that are developed for the hospital as an institution, often almost meeting with ethics of biomedicine. It can also be said that the ethics of an institutional hospital entails the development of the ethics of biomedicine (bioethics) because of new ethical problems or dilemmas, such as the impact or consequence of the rapid progress of science and technology in biomedicine [4].

According to the Islamic faith, human life should be beneficial and, as much as possible, humans should do good, as stated in the Qur'an Surah Al-Ashr (verses 1–3). Allah says, “*By time,* (1) *Indeed, mankind is in loss,* (2) *Except for those who have believed and done righteous deeds and advised each other to truth and advised each other to patience.*” Shariah hospitals should provide the best health services, more than the patient expects. The Prophet stated that the most beloved man of God benefits higher than others, and the most beloved work of God is to make others happy and alleviate hardship [5]. Healthcare at hospitals do so with the appropriate standard of quality of services that can benefit the patient. Similarly, in the Qur'an Surah Ash-Sharh (verses 6–8), Allah says, “*Indeed, with hardship [will be] ease.* (6) *So when you have finished [your duties], then stand up [for worship]*. (7) *And to your Lord direct [your] longing* (8).” On this occasion, providing healthcare (with hardship) is not only measured by finishing the duties but also done appropriately according to the standard of quality of services while longing to the God [6].

Hospitals with Shariah certification carry out their services with reference to the concept of *Maqoshid Al-Sharia*, namely, the preservation of religion (*Hifzh Al-Din*), the soul (*Hifzh Al-Nafs*), the senses (*Hifzh Al-‘Aql*), the descent (*Hifzh Al-Nasl*), and property (*Hifzh Al-Mal*) [3]. As commanded by Allah in the Qur'an surah Jaatsiyah, verse 18, “*Then We made you on top of a sharia (laws) of matter (religion it). Then follow the Shari'a, and do not follow the desires of lust people do not know.*” According to the verse, the implementation of health services should be based on Sharia to ensure the good of the world and the hereafter.

Maqoshid Al-Sharia is the value or spirit contained in Sharia law. Sharia is a set of rules set by Allah for his creatures to be used as a guide in regulating relations with the creator as well as relations between humans and all of nature [7]. Sharia also refers to all the laws that Allah established for all humans to be believed and carried out for their own benefit, both in this world and hereafter [8]. According to the word of God in Surah al-Maidah (verse 48), “*And We have revealed to you*, *[O Muhammad]*, *the Book in truth, confirming that which preceded it of the Scripture and as a criterion over it. So judge between them by what Allah has revealed and do not follow their inclinations away from what has come to you of the truth. To each of you, We prescribed a law and a method. Had Allah willed, He would have made you one nation [united in religion], but [He intended] to test you in what He has given you*; *so race to [all that is] good. To Allah is your return all together*, *and He will [then] inform you concerning that over which you used to differ.*” Thus, the application of Sharia principles in hospital services aims to benefit humans both alive and deceased. The application of *Maqoshid Al-Sharia* in hospital services implies that Shariah hospitals are responsible for five things: they must protect their patients in terms of their beliefs, protect their patients' rights for being saved, protect their patients from wasting wealth, protect their patients for the maintenance of common sense, and protect their patients for the good of their offspring [3].

The Tangerang General Hospital, as with any government hospital, is in the process of meeting certification as a Shariah hospital to serve the population in the city of Tangerang, of which the majority practices the religion of Islam. Such determination to meet this certification is a challenge for the hospital management, which consists of health personnel from different religions and tribes. All parties should implement the principles of Sharia on all health services, so that such services would be optimally accomplished. Hence, the study of the application of the ethical code of Shariah hospitals is important in increasing health personnel performance, which can assist the decision making of the Tangerang General Hospital management.

## 2. Methods

### 2.1. Study Design

The research design used in this study is quantitative analysis, with the retrieval of cross-sectional data in the months of July–August 2019 at the Tangerang General Hospital.

### 2.2. Participants

Population studies in the field of health have included doctors, nurses, and other health professionals. This research uses a sample of 119 health professionals.

### 2.3. Data Collection

The study uses simple random sampling and a questionnaire answered with the consent of the respondents. The questionnaire in this study was developed from the Guide Book of Ethical Code of Shariah Hospital, issued by the Islamic Health Institution Network of Indonesia (MUKISI; https://www.mukisi.com/). This instrument was also adjusted according to Guideline for Shariah Hospital Implementation, no. 107/DSN-MUI/X/2016, issued by *fatwa* of The National Sharia Board–Indonesian Council Ulema, formerly chaired and signed by Prof. Dr. KH. Ma'ruf Amin, Vice President of Indonesia 2020–2024 (Supplementary 1).

### 2.4. Data Analysis

**Figure 1:**
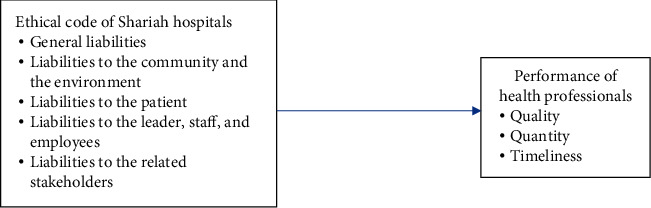
Research concept.

The method of data analysis used in this research is structural equation modeling (SEM). This can determine whether the ethical code of Rumah Sakit Sharia has an influence on the performance of health personnel, how significant this influence is, and how significant the two variables are. For the ethical code of Shariah hospitals, four obligations and one hospital association with stakeholders are highlighted (Figure 1).

The variables used to determine the respondents' answers to the questionnaire are described. An analytical index is used to determine the propensity of these answers to each variable, which will be based on the values of the score average, categorized based on the calculation of the three-box method [9].

  Limit on the range of the score variable on the ethical code of Shariah hospitals and the performance of health workers: (% *F* × 4)/4 = (119 × 4)/4 = 119.
  Limit under the range of the score variable on the ethical code of Shariah hospitals and the performance of health workers: (% *F* × 1)/4 = (119 × 1)/4 = 29.75.
  The figures and indices produced showed a score of 29.75–119, with a range of 89.25. Using the three-box method, the range is divided into three parts, which yields the range for each section at 29.75 as follows:
  29.75–59.50: low
  59.51–89.35: moderate
  89.36–119: high
  Mechanical scoring is used to score a maximum of 4 and a minimum of 1. Then, the calculation of the index answers of the respondent is done with the following formula:
  Value index = [(%*F*1 × 1) + (%*F*2 × 1) + (%*F*3 × 1) + (%*F*4 × 1)]/4

## 3. Results

The majority of the health force is women (80%) and has not yet married (75.8%) (Table 1).

### 3.1. Ethical Code of Shariah Hospitals

As given in Table 2, the average value for the variable of the ethical code of Shariah hospitals is 95 (category: high). This means that the respondents, as health workers, have a positive perception toward the implementation of this ethical code at the Tangerang General Hospital. The index highs are indicators of the obligations of Shariah hospitals, with the value of the index amounting to 97 (category: higher). This indicates that the health workers at the Tangerang General Hospital have already attempted to apply the values of Maqoshid Al-Sharia Al-Islamiyah in providing health services.

As given in Table 3, the average value for the variable of the performance of health workers at the Tangerang General Hospital is 86.417 (category: medium). This means that according to the respondents, the hospital does not optimally provide health services. The index highs are indicators of the quality of the service results, with the value of the index amounting to 91.5 (category high). Healthcare at the Tangerang General Hospital is carried out according to the standard of service quality, focusing on service to the patient (patient*-*centeredness), and the impact expected is the improved well-being of the patients and the health workers.

**Figure 2:**
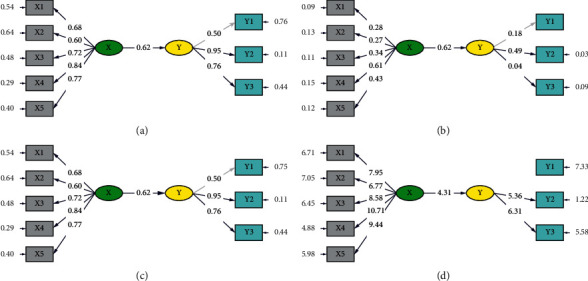
(a) Conceptual diagram. (b) Estimated value diagram. (c) Standardized solution diagram. (d) *T* values. X, ethical code of Shariah Hospital; Y, performance power of the Tangerang General Hospital; X1, general obligations of the Shariah Hospital; X2, obligations of the hospital to society and the environment; X3, obligations of the hospital to patients; X4, obligations of the hospital to leaders, staff, and employees; X5, obligations of the hospital to related institutions; Y1, quality of health services; Y2, quantity of health services; Y3, timeliness (accuracy) of health services.

The diagram shows that the quality, quantity, and timeliness (accuracy) of health services are indicators of the variable of health professional performance at the Tangerang General Hospital (Figure 2(a)). From the SEM analysis, all the indicators have been significant. This can be seen from the *t* value of each indicator being greater than 1.658 (*t* value 5%, with the data amounting to 119 samples). For example, the indicator of general liabilities has not amounted to 7.95, while that of liabilities to society and the environment has a *t* value 6.77, that of liabilities to patients has a *t* value of 8.58, that of liabilities to the leader, staff, and employees has a *t* value of 10.71, and that of obligations to related institutions has a *t* value of 0.044 < 1.658 (not significant). Likewise, the indicators of quality, quantity, and timeliness all have a *t* value greater than the *t-*count of the *t* value table; the largest is the indicator of the quantity of services, amounting to 5.36.

### 3.2. Diagram Path

The results obtained in the path diagram are the same as those of the SIMPLIS output. The output path diagram is shown in Figures 2(b) and 2(c). The displayed path diagram shows the value of the estimated and unstandardized relationships among the variables.

The value estimates are equal to the output shown in Figure 2(b), while the value of the covariance between variable latencies can be seen in the numbers in the arrows. The output shows the correlation values of the matrix of covariance between the two variable latencies. The covariance between the ethical code of Shariah hospitals and the performance of health professionals at the Tangerang General Hospital amounted to 0.62.

Figure 2(c) shows the value of a standardized relationship among the parameters. The output above shows that the value of a standardized indicator of the liability to the leader, staff, and employees provide the largest influence to the variable of the ethical code of Shariah hospitals (0.84), while the variable of health professionals' performance had the largest influence on the indicator of the quantity of performance (0.95).

The output path diagram shown in Figure 2(d) displays the *t* values for the estimation of the parameters. The relationships are significant at 5% (default Lisrel). The relationship between the ethical code of Shariah hospitals and the performance of health professionals is significant as *t* values = 4.31 (loading factor > 1.960). The ethical codes of hospitals guide the organizations in directing ethical health personnel behavior as well as guidelines for decision making. Thus, such a code is important to the improvement of health worker performance.

## 4. Discussion

This study reveals that there is a significant relationship between ethical code of Shariah hospitals and the performance of health professionals. An effective and healthy work environment can be achieved by maintaining high standard professional integrity as well as treating each personnel with respect and fairly [10]. Furthermore, a more beneficial work circumstance may be also attained without intimidation, abusive, or offensive behavior of conduct [11]. Creating warranted tolerance between employees can also bring a positive way to encourage employers. In today's corporate culture, the importance of ethics and moral values has become an organizational standard, which has defined that business achievement measures much more than profit margins. In the genuine value, the company successfully determined the image and the goodwill it conducts [12]. Hence, top and brilliant workforce will be attracted to such companies. Previous experiences have proven a relationship between ethical conduct and job satisfaction [13–15]. Since providing an ethical environment can produce productive employees due to their satisfaction, this standard of workplace can bring an adequate sense of belonging and loyalty as one of the substantial motivating aspects. It is no doubt that there is an investment if a company has the high ethical standards as well as a liability as its ethical conduct [16]. Thus, the implementation of ethical code, which enforces performance in this study, particularly on health workers through Shariah's code of conduct, is essential to establish.

Organizational culture, as perceived by the public, is owned by all the members of the organization. So any employee who becomes a member of the organization will have to exhibit values, beliefs, and behavior in accordance with the organization [17]. This can be likened to a code of ethics at an institute or hospital; when this is implemented, all the health and nonhealth professionals will exhibit the values, beliefs, and behavior in accordance with the code of ethics of the profession.

The impact of the ethical code has been extensively acknowledged in providing the effectiveness including improving responds to the issue that customers care about [18]. Its effectiveness not only produce balance and benefit but also has more values at work [19]. Conversely, absence of the codes of conduct, which should be set as a uniform of ethical guidelines and application, will interrupt and make a failure to deliver services to the customers in a work environment [20]. Even though it has been drafted, the undetailed standard and unsupported policies would be challenging the effectiveness of work circumstance, particularly in a complex system such as hospital [21].

The implementation of a code of ethics at a hospital requires commitment then requires periodical socialization and education for all levels of health and nonhealth professionals (a minimum of one program per year), including adequate public health information system management [22]. The code of ethics of an organization/hospital must also adapt to the developments and regulations of the institute related to the force [23]. In the end, the implementation of Shariah approach as a code of ethics at a hospital can create a proper and respectable culture. Furthermore, a study about Shariah hospital's code of conduct by presenting its beneficiary to not only improve health personnels' performance but also to provide the inclusive health service for all religions and cultures is essential.

## 5. Limitation

Our study presents new insight about the implementation of the ethical code of Shariah hospitals and the improvement of health personnel performance, but it has certain limitations. First, since we employed cross-sectional approach as one-shot time assessment to present the ethical code of Shariah hospitals, four obligations, and one hospital association with stakeholders, this study was not adjusting the cause-effect between the two variables. However, this study is presented by path analysis of SEM which can generalize the relationship between two variables as well as present the reality condition as added values of the implementation of ethical code of Shariah with health professionals' performance in a hospital. Second, the SEM calculation was also lacking to set control variables since there are many variables that affect performance in addition to ethics codes. However, this study was using the SIMPLIS method of SEM by bridging and calculating the measurement equation between latent variables, so it can explain the comparability among these variables.

## 6. Conclusion

The establishment of the ethical code of Shariah hospitals, whether explicit or implicit, can directly improve the performance of all health workers at the Tangerang General Hospital. As such, the synergy between the hospital and the health workers can improve the performance of both levels of individuals as well as that of the hospital. The leaders and health workers at the Tangerang General Hospital are highly expected to remain consistent and committed to implementing all the obligations set out in the ethical code of Shariah hospitals, with the principal objective of maintaining the safety of the patients.

## Figures and Tables

**Table 1:** Individual characteristics.

No.	Factor		*N*	Percentage
1	Gender	Male	24	20
		**Female**	**95**	**80**
2	Marriage status	**Not married**	**90**	**75.8**
		Married	29	24.2
3	Education	Diploma	8	6.7
		**Bachelor**	**71**	**60**
		Profession (MD, pharmacist, nurse, and so on)	26	21.7
		Specialist (pediatrician, cardiologist, and so on)	14	11.7

**Table 2:** Description score index of the ethical code of Shariah hospitals at the Tangerang General Hospital.

Variable	Indicator	Score				Amount	Index	Category
		1	2	3	4			
Ethical code of Shariah hospitals	**General liabilities**	0	1	86	32	119	**97**	**High**
		0	2	258	128	388		
	Liabilities to society and the environment	0	8	87	24	119	93.25	High
		0	16	261	96	373		
	Liabilities to the patient	0	10	77	32	119	94.75	High
		0	20	231	128	379		
	Liabilities to health personnel	3	12	67	37	119	94	High
		3	24	201	148	376		
	Liabilities to associated institutions	1	6	77	35	119	96	High
		1	12	231	140	384		

**Amount**							**475.00**	**High**
**Average**							**95**	

**Table 3:** Description of health workforce performance index scores at the Tangerang General Hospital.

Variable	Indicator	Score				Amount	Index	Category
		1	2	3	4			
**Health workforce performance**	**Quality**	0	1	108	10	119	**91.5**	**High**
		0	2	324	40	366		
	Quantity	0	23	77	19	119	88.25	Moderate
		0	46	231	76	353		
	Timeliness	0	42	74	3	119	79.5	Moderate
		0	84	222	12	318		

**Amount**							**259.25**	**Moderate**
**Average**							**86.417**	

## Data Availability

The datasets generated and/or analysed during the current study are not publicly available due to confidentiality, but are available from the corresponding author upon request.

## Abbreviation

SEM;: Structural equation modeling.

## Supplementary Materials

Supplementary Materials Questionnaire of the study and its guide.
