# Supplementary material for: Health Personnel Improvement in the Implementation of Shariah's Ethical Code of Conduct in Tangerang Hospital, Indonesia
Source: J Environ Public Health. 2022 May 9;2022:5548840. doi: 10.1155/2022/5548840 (PMC9110242; doi:10.1155/2022/5548840)
Supplement: Supplementary Materials — Questionnaire of the study and its guide. [file 5548840.f1.docx]

Supplementary 1. Questionnaire of the study (translated from Bahasa)

**Questionnaire**

The questionnaire in this study was developed from the Guide Book of Ethical Code of Shariah Hospital, issued by the Islamic Health Institution Network of Indonesia (MUKISI; https://www.mukisi.com/). This instrument was also adjusted according to Guideline for Shariah Hospital Implementation, No. 107/DSN-MUI/X/2016 (<https://ubico.id/wp-content/uploads/2019/02/107-Pedoman-Penyelenggaraan-Rumah-Sakit-Syariah.pdf>), issued by *fatwa* of The National Sharia Board – Indonesian Council Ulema - formerly chaired and signed by Prof. Dr. KH. Ma’ruf Amin; Vice President of Indonesia 2020-2024.

Dear Nurse/Sir/Madam (Doctor, Nurse, and other Health Professionals)

*Assalamu’alaikum warahmatullahi wabarakatuh*

In order to improve productivity of health personnel in Tangerang General Hospital leading hospital management Shariah, we will conduct a survey on the Code of Islamic Hospital to health workers performance. The results of this survey will not affect the assessment of health workers at the hospital. We ask you to answer all the questions according to the actual conditions in the hospital. All answers are correct there are no wrong answers. Your answers will be kept confidentially according to research ethics. The completeness of your answers is very important and affects the survey results. This as will directly assist the implementation of the Code of Conduct Sharia Hospital.

Thank you for your attention and cooperation

*Wassalam,*

Best Regards,

Wahyu Sulistiadi

(Team Researchers Coordinator)

1. **IDENTITY**
   1. Name (Initials) :
   2. Age : …years
   3. Gender : ( ) Male ( ) Female
   4. Working Period : …years
   5. Status Marriage :
   6. Education Level :
   7. Installation/Unit/Section :

1. **CHARGING INSTRUCTIONS**
2. Choose the answer that is in accordance with the opinion of Sir/Madam by giving a check list (V) in the place where applicable.

1. Answers should be in accordance with real conditions in the hospital, with the following rules:

Strongly Disagree  = STS

Disagree  = TS

Agree  = S

Strongly Agree  = SS

1. **INDIVIDUAL CHARACTERISTICS**

| **NO** | **STATEMENT** | **STS** | **TS** | **S** | **SS** |
| --- | --- | --- | --- | --- | --- |
|  | **Interest** |  |  |  |  |
| 1 | I work optimally to utilize the existing facilities & infrastructure (facilities) in the Sharia Hospital so that it can support the safeguarding of my religion. |  |  |  |  |
| 2 | I feel comfortable working in a Sharia Hospital so that my religion, soul and mind can be maintained. |  |  |  |  |
| 3 | Hospital Management Sharia can help me in order to be able to work with colleagues within and between the service unit. |  |  |  |  |
|  |  |  |  |  |  |
|  | **Personality** | **STS** | **TS** | **S** | **SS** |
| 1 | I only carry out work that is in accordance with the Maqoshid Al-Sharia rules. |  |  |  |  |
| 2 | Every problem seems to contain difficulties, therefore if I face a problem then I try to solve it with the Maqoshid Al-Sharia rules. |  |  |  |  |
| 3 | My goal at work is the development of self-potential and increasing knowledge in the framework of guarding my intellect (Hifzh Al'aql). |  |  |  |  |
|  |  |  |  |  |  |

1. **SHARIAH HOSPITAL CODE OF CONDUCT**

| **NO** | **STATEMENT** | **STS** | **TS** | **S** | **SS** |
| --- | --- | --- | --- | --- | --- |
|  | **General Obligations of Sharia Hospitals** |  |  |  |  |
| 1 | The hospital has a true and honest vision, mission and management of health services. |  |  |  |  |
| 2 | Hospital leaders convey strategies for achieving the vision, mission and goals of the hospital. |  |  |  |  |
| 3 | I am in duty is always to apply the values mandate (unbelievable). |  |  |  |  |
| 4 | Health workers are encouraged to be innovative in doing work by prioritizing quality services according to the Minimum Service Standards and Minimum Service Standards for Sharia Hospitals. |  |  |  |  |
| 5 | Health service support facilities greatly support security (safety) for health workers and patients. |  |  |  |  |
| 6 | The quality of health personnel services in the hospital has an effect on patient satisfaction which has an impact on the care of the patient's soul, mind, property, ancestry and religion. |  |  |  |  |
| 7 | Health workers have the opportunity to learn to follow developments in hospital science or according to the knowledge of each health professional profession. |  |  |  |  |
| 8 | The hospital environment is very conducive to encourage health workers to document all health service activities. |  |  |  |  |
| 9 | The storage and maintenance of medical records is well done in the hospital. |  |  |  |  |
| 10 | Health workers are encouraged to take reasonable risks (for example, trying new ideas, new ways of doing things) by sticking to the Sharia Hospital Standards. |  |  |  |  |
|  |  |  |  |  |  |
| **NO** | **STATEMENT** | **STS** | **TS** | **S** | **SS** |
|  | **Hospital Obligations to Society and the Environment** |  |  |  |  |
| 1 | All health workers in the hospital have a health promotion program in the hospital community. |  |  |  |  |
| 2 | The hospital has a good Environmental Impact Analysis (*AMDAL*) management according to the rules. |  |  |  |  |
| 3 | The hospital has a health coaching program in the village/sub-district/sub-district of the hospital's target area. |  |  |  |  |
| 1. Answers should be in accordance with real conditions in the hospital, with the following rules:   TP : Never been done  JD : Rarely Done  SD : Always one (never done occasionally)  SR : Do it often | | | | | |
| **NO** | **STATEMENT** | **TP** | **JD** | **SD** | **SR** |
|  | **Hospital Obligations Toward Patients** |  |  |  |  |
| 1 | I invite patients to read *Basmallah* before the procedure and take medication. |  |  |  |  |
| 2 | I suggest that patients wear a hijab during their hospital stay. |  |  |  |  |
| 3 | I provide education about *taharah*to patients. |  |  |  |  |
| 4 | I provide education about *prayer*to patients |  |  |  |  |
| 5 | I provide education about *talqin* to patients and their families |  |  |  |  |
| 6 | The hospital provides spiritual books to patients. |  |  |  |  |
| 7 | The TV program in the hospital has Islamic education. |  |  |  |  |
| 8 | Health workers provide information and perform EKG placement according to gender |  |  |  |  |
| 9 | Health workers always provide information related to wearing the hijab in the operating room to patients. |  |  |  |  |
| 10 | Health workers always provide education for breastfeeding mothers to still wear the hijab. |  |  |  |  |
| 11 | The hospital informs us about the operation schedule which does not interfere with prayer times. |  |  |  |  |
| 12 | Health workers provide information / socialization about talqin to patients / patient families |  |  |  |  |
| 13 | The hospital chants the call to prayer every 5 prayers |  |  |  |  |
| 14 | I remind the patient of prayer times |  |  |  |  |
| 15 | Health workers provide information about catheter placement according to gender/family companion |  |  |  |  |

| **NO** | **STATEMENT** | **TP** | **JD** | **SD** | **SR** |
| --- | --- | --- | --- | --- | --- |
|  | **Hospital Obligations for Health Workers** |  |  |  |  |
| 1 | I can carry out tasks with my own effort/way without having to harm the hospital while maintaining the patient's religion, soul, mind, ancestry and assets. |  |  |  |  |
| 2 | Hospital management guarantees occupational health and safety for employees, especially health workers. |  |  |  |  |
| 3 | Hospital management creates a conducive atmosphere to ensure the creation of the unity (*ukhuwah*) Islamiyah between the leadership, staff and employees. |  |  |  |  |
| 4 | Hospital management always fosters leaders, staff and employees with Islamic values. |  |  |  |  |
| 5 | The compensation received by health workers is in accordance with performance achievement. |  |  |  |  |

| **NO** | **STATEMENT** | **TP** | **JD** | **SD** | **SL** |
| --- | --- | --- | --- | --- | --- |
|  | **Hospital Obligations with Related Institutions** |  |  |  |  |
| 1 | Hospital management always maintains a good relationship with the owner according to mutual advice principles of truth and patience. |  |  |  |  |
| 2 | Hospital management maintains good relations with the Indonesian Ulema Council, government agencies or other bodies engaged in the health sector. |  |  |  |  |
| 3 | Hospital management tries to maintain good relations between hospitals with the principle of competing for goodness. |  |  |  |  |
| 4 | Hospital management tries to be a place for the education of health and medical personnel. |  |  |  |  |

| **NO** | **STATEMENT** | **TP** | **JD** | **SD** | **SR** |
| --- | --- | --- | --- | --- | --- |
|  | **Work productivity** |  |  |  |  |
| 1 | My work results are increasing because of the competencies (skills, knowledge and work attitudes) that I have. |  |  |  |  |
| 2 | I was unable to reach the target set by the hospital. |  |  |  |  |
| 3 | I cannot finish my job on time. |  |  |  |  |
| 4 | My work has met the requirements set by the hospital. |  |  |  |  |
| 5 | I finish my work according to the scheduled time. |  |  |  |  |
| 6 | I feel that my work is not in accordance with the competencies I have. |  |  |  |  |
| 7 | I use work time for other purposes. |  |  |  |  |
| 8 | When compared to the past before the Sharia Hospital policy, my work results have increased. |  |  |  |  |
| 9 | I want to make the right strategy in getting the job done. |  |  |  |  |
| 10 | I don't want to make the same service mistakes over and over again. |  |  |  |  |
| 11 | The Sharia Hospital code of ethics helps me to provide optimal health services. |  |  |  |  |
| 12 | I use my work experience to improve my work. |  |  |  |  |

**THANK YOU**

1
